# A Comparison of Approaches for Motion Artifact Removal from Wireless Mobile EEG During Overground Running

**DOI:** 10.3390/s25154810

**Published:** 2025-08-05

**Authors:** Patrick S. Ledwidge, Carly N. McPherson, Lily Faulkenberg, Alexander Morgan, Gordon C. Baylis

**Affiliations:** 1 Department of Psychological Sciences, Western Kentucky University, 1906 College Heights Blvd., Bowling Green, KY 42101, USA; carly.mcpherson782@topper.wku.edu (C.N.M.); lily.faulkenberg879@topper.wku.edu (L.F.); gordon.baylis@wku.edu (G.C.B.); 2 Department of Allied Health, Sport & Wellness, Baldwin Wallace University, 275 Eastland Rd., Berea, OH 44017, USA; amorgan@bw.edu

**Keywords:** electroencephalography (EEG), event-related potentials (ERP), mobile brain imaging, signal processing, independent components analysis (ICA)

## Abstract

Electroencephalography (EEG) is the only brain imaging method light enough and with the temporal precision to assess electrocortical dynamics during human locomotion. However, head motion during whole-body movements produces artifacts that contaminate the EEG and reduces ICA decomposition quality. We compared commonly used motion artifact removal approaches for reducing the motion artifact from the EEG during running and identifying stimulus-locked ERP components during an adapted flanker task. EEG was recorded from young adults during dynamic jogging and static standing versions of the Flanker task. Motion artifact removal approaches were evaluated based on their ICA’s component dipolarity, power changes at the gait frequency and harmonics, and ability to capture the expected P300 ERP congruency effect. Preprocessing the EEG using either iCanClean with pseudo-reference noise signals or artifact subspace reconstruction (ASR) led to the recovery of more dipolar brain independent components. In our analyses, iCanClean was somewhat more effective than ASR. Power was significantly reduced at the gait frequency after preprocessing with ASR and iCanClean. Finally, preprocessing using ASR and iCanClean also produced ERP components similar in latency to those identified in the standing flanker task. The expected greater P300 amplitude to incongruent flankers was identified when preprocessing using iCanClean. ASR and iCanClean may provide effective preprocessing methods for reducing motion artifacts in human locomotion studies during running.

## 1. Introduction

Recent advances in electroencephalography (EEG) and signal processing have led to the development of EEG-based mobile brain–body imaging approaches to characterize central nervous system dynamics in human locomotion [1,2,3,4,5,6]. Historically, the main barrier to progress in the scientific understanding of the neurophysiology of human locomotion has been the presence of the mechanical/non-physiological artifact in the EEG time series due to head motion, electrode displacement, and/or cable sway. Such artifacts will be typically time-locked to the gait cycle. Many have begun to optimize signal processing tools to remove this artifact from the EEG during walking, while seeking to minimize degradation of the ground-truth signal.

Independent components analysis (ICA) is a commonly implemented blind source separation approach that uses linear decomposition of EEG from multiple electrodes to identify maximally independent components in the data, such as those of physiological origin [7,8,9]. Many have also used ICA approaches to identify the gait-related artifact in human walking [3,10,11,12,13] and have characterized walking-related EEG spectral power changes in independent components of cortical origin. Following ICA decomposition, ICLabel [14] is a common EEGLAB plugin [7] used for classifying the independent components as brain or artifactual (e.g., eye, heart, muscle, line noise) and is based on a large dataset of labeled components by users. However, ICLabel has not been trained on mobile EEG data, does not adapt to the present dataset, and the continued presence of large motion artifacts may contaminate the ICA’s ability to identify maximally independent sources [15]. Rather than attempting to parse out motion artifacts through ICA, end users may choose to reduce or eliminate motion artifacts during preprocessing and prior to ICA.

### 1.1. Artifact Subspace Reconstruction

Beyond blind source separation methods, artifact subspace reconstruction [16,17] and iCanClean [1,2,18] have recently been applied in human studies of walking for motion artifact removal. (Readers are directed to [6,15] for more scoping reviews). Artifact subspace reconstruction (ASR) uses principal components analysis to identify high-amplitude artifacts in continuous EEG based on a baseline calibration period and a standard deviation threshold for artifact identification based on the distribution of signal variance [19,20]. ASR’s performance in identifying high-amplitude artifacts, such as those produced by motion, is dependent on its method for selecting the periods that make up the calibration ‘reference’ data. First, the root mean squares (RMS) of sliding windows of 1 s segments are calculated from the continuous EEG time series. A condensed Gaussian distribution is used to convert the RMS values into z-scores [20].

The data segments that make up the reference data are those with z-scores that fall within −3.5 to 5.0 of the Gaussian distribution for at least 92.5% of electrodes within that segment [19,20]. Given these parameters, such data segments are assumed to be free of outliers and thus make up the reference data that is used to interpolate the scalp-recorded data based on its covariance matrix. Next, a sliding-window principal components analysis (PCA) derives principal components on the reference data, the RMS for each component is calculated, and the mean and standard deviation for all RMS values are calculated across the reference period. The non-reference data undergo PCA similar to the reference data and principal components and are identified as artifactual if its standard deviation of RMS exceeds the user-defined threshold (“k”) and the time series is reconstructed based on the calibration data. Independent sources of EEG activity are dipolar [21] and component dipolarity is often used to evaluate the quality of ICA decomposition.

In non-locomotion studies, ASR has been shown to reduce eye and muscle artifacts and improve the dipolarity of ICA decompositions; the extent to which ASR negatively impacts non-artifactual dipolar brain sources is based on the user-defined component threshold, and a k threshold of 20–30 has previously been recommended [19], with a lower threshold producing larger data manipulations. When human walking preprocess was studied using ASR, ICA decompositions produce the most dipolar and reproducible components if the k parameter does not fall below 10 [22], because if k is too low, it is possible to ‘overclean’ one’s data and inadvertently manipulate the intended signal [6]. Very recently, limitations in ASR’s algorithm for identifying the reference period have been reported which may explain why higher recommended k values may fail to address high-amplitude artifacts produced by motion [20]. Our choice of k value was made with a view of being aggressive, while avoiding “over-cleaning” the data. We return to this point in the discussion.

### 1.2. iCanClean

ICA decomposition and component dipolarity has been shown to be improved using iCanClean [1,2,18]. iCanClean leverages reference noise signals and canonical correlation analysis (CCA) to detect and correct noise-based subspaces on a user-selected criterion (R^2^) of the correlation between the subspaces of corrupt EEG and noise signals [1,2,18]. iCanClean is ideally implemented with noise sensors that are mechanically coupled to electrodes but only capture noise, such as those due to motion, because they are not in contact with the scalp [1,2]. Since the EEG recorded from scalp electrodes contains a mixture of signal and noise, and the ‘dual-layer’ noise sensors only contain noise, it is possible to subtract subspaces of noise from the scalp EEG [1,2,5]. When dual-layer sensors are not available, ‘pseudo-reference’ noise signals are created from the raw EEG by temporarily applying a user-selected notch filter to identify noise within the EEG, such as below 3 Hz, for example. Canonical correlation analysis (CCA) identifies subspaces of the scalp EEG that are correlated with subspaces of noise (e.g., motion artifact subspaces). This is based on user-selected criterion of the correlation of scalp EEG subspaces with noise subspaces (R^2^) and the sliding time window(s) used to execute the CCA. After the noise components are projected back onto the EEG channels using a least-squares solution, the noise components that exceed the R^2^ threshold are subtracted from the scalp EEG [1,2,18].

Using a phantom head model, iCanClean outperformed ASR in identifying and correcting artifactual sources, including the simulated walking artifact, and reproducing EEG that most closely resembled ground-truth signals [1]. In non-simulated human locomotion data during walking, it was shown that an R^2^ of 0.65 and sliding window of 4 s produced the most dipolar brain components from ICA [2]. iCanClean using dual-layer noise electrodes has recently been used to identify electrocortical activity during walking in realistic scenarios, such as when walking on uneven terrain [23]. These mobile brain–body imaging EEG studies and others have advanced the understanding of the role of sensorimotor alpha/beta suppression in walking [24,25] and increased prefrontal beta/theta power during gait adaptations [13,23,26].

### 1.3. Current Study

Electrocortical dynamics that regulate human locomotion during running are currently unknown, likely because few investigations of running-related motion artifact removal have been published [3,10]. Running, particularly overground, tends to produce broadband spectral power, particularly at the step frequency and its harmonics, which can be minimized but not eliminated using ICA-based motion artifact removal approaches [3]. Furthermore, although iCanClean has been shown to be effective in reducing walking-related motion artifact using dual-layer electrodes, it is currently unknown how this approach performs with pseudo-reference signals. Finally, although iCanClean produces more dipolar sources than ASR in phantom head data with simulated walking artifacts [1], how these two approaches compare in addressing the motion artifact during human running is unknown. Some have shown that preprocessing mobile EEG with both single-and dual-layer ASR and iCanClean can capture event-related spectral perturbations time-locked to the gait cycle [24], and ASR appears to effectively handle low-density multichannel EEG in preliminary non-locomotion studies [27,28].

The overarching purpose of the present study was to evaluate artifact subspace reconstruction and iCanClean approaches for motion artifact removal from mobile EEG during overground running. We introduce a novel dynamic adaptation of the Flanker task [29,30] that would allow us to examine how these artifact removal approaches affect (a) ICA-derived component dipolarity [2], (b) spectral power at the frequency of the gait artifact and its harmonics [3], and (c) stimulus-locked event-related potential (ERP) components [3].

We examined whether iCanClean or ASR would produce the most dipolar brain components [1]. We also examined how iCanClean and ASR would vary in their handling of the motion-related artifact at the frequency of the gait artifact, and ERP components similar in time course to a standing flanker task without whole-body movement. The standing flanker task mirrored the dynamic flanker task, but the absence of motion ensured that no motion artifact would be present. Finally, we evaluated whether the P300 congruency effects expected in this standing flanker task could be identified in the dynamic flanker task after implementing ASR and/or iCanClean. Optimizing signal processing methods for handling motion artifacts during ecologically valid running paradigms would aid in understanding the electrocortical dynamics, and their relationships to lower-extremity biomechanics, that support running.

## 2. Materials and Methods

### 2.1. Participants

The sample included 21 young adult (18–30 years) athletes (TAS ≥ 5) [31] with a history of playing sports with running and lateral movements (e.g., soccer, basketball, lacrosse). Eligibility included an absence of prior lower extremity injury, brain injury, or neurodevelopmental disorder. Non-corrected or corrected vision for all participants was 20/25 or better. All participants completed 10 min of eyes-open resting state followed by a novel dynamic flanker task (150 min), which included an overground jog and sidestep maneuver (E-prime 3.0, Psychology Software Tools, Pittsburgh, PA, USA) during wireless 16-channel EEG recording (Mentalab, Munich, Germany). Nine of these participants also completed a standing flanker task (30 min). At the conclusion of the study, participants were compensated with course credit or a $40 gift card.

### 2.2. Dynamic Flanker Task

**Figure 1:**
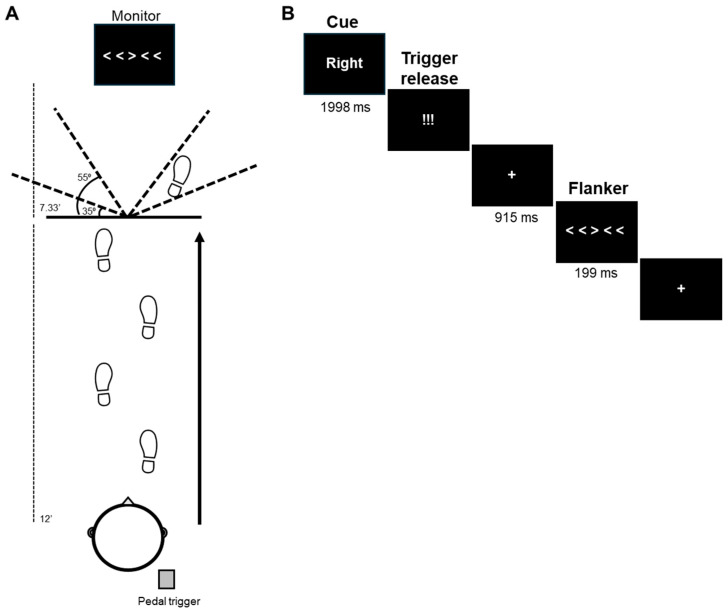
EEG dynamic flanker task. (**A**) During mobile EEG recording, participants jog and perform a change in direction (sidestep) at the end of the 12-foot runway based on the direction of the middle arrow of the flanker stimulus. Trial types varied based on anticipation (cued, not cued), flanker arrow congruence (congruent, incongruent), and direction (left, right). (**B**) In this example trial, the right–incongruent flanker is cued. Flanker onset is 915 ms after trigger release.

The trial sequence is illustrated in Figure 1. Each trial began with the participant in a staggered running stance, centrally positioned at the start of a 12-foot-long runway, and with their right heel pressed down on a foot pedal trigger (Chronos; E-Prime 3.0; Psychology Software Tools, Pittsburgh, PA, USA) and their left foot in front of them (Figure 1A). The back of the participant’s right heel was positioned 2 feet from the start of the runway and the front aspect of the left foot was directly behind the start of the runway. Participants were instructed to run forward at a consistent pace (4.5 ± 10% mph), maintain attention on a 27″ monitor (7.33 ft after end of runway), and perform a left or right 35–55 degree sidestep [32,33] at the end of the runway based on a visual flanker stimulus. The middle arrow of the flanker stimulus [29,30] indicated the direction of the sidestep for that trial. Participant’s anticipation of the sidestep direction was manipulated via the presentation of a cue at the beginning of the trial.

Each trial began with the participant’s right heel on the foot pedal and a “Ready”? (64 pt) screen (1998 ms) at central fixation (Figure 1B). Following, a cue (84 pt) was presented (1998 ms). For anticipated trials, the cue given was “Left” or “Right” which matched the direction of the forthcoming middle arrow of the flanker stimulus. In unanticipated trials, no cue was provided (i.e., “XXXXX”). After the cue, participants were presented with a stimulus (“!!!”; 110 pt font) which instructed them to begin jogging. The participant’s release of the foot pedal triggered the presentation of the flanker stimulus 915 ms later. This constant foreperiod was chosen to be consistent with other response pre-cueing tasks [34,35,36]. A central fixation cross (48 pt font) was presented during the foreperiod.

The flanker stimulus (172 pt font) was presented for 199 ms [30,37,38]. The middle arrow was either congruent (> > > > > ; < < < < <) or incongruent (< < > < < ; > > < > >) to the other arrows [30,38,39] and determined the direction of the sidestep maneuver for that trial. Participants were instructed to ignore the flanker arrows and perform the sidestep maneuver in the direction of the middle arrow. The flanker stimuli subtended an approximate visual angle of 7.74 degrees (horizontal) × 0.99 degrees (vertical) when it appeared as viewed from halfway down the runway. All stimuli were presented in white Times New Roman font on a black background. After the end of the trial, the participant slowly walked back to the start of the runway.

Participants completed 8 blocks of 24 trials over a period of 60–80 min. Trials varied by anticipation (anticipated vs. unanticipated via the cue), sidestep direction (left vs. right via the flanker stimulus), and flanker congruence (congruent vs. incongruent). Each block included random and equal presentations of the four stimulus types (< < < < <, > > < > >, > > > > >, < < > < <) in anticipated and unanticipated conditions. Prior to the experimental trials, participants completed a light plyometric warm-up and one block of 12 trials in each of the anticipated and unanticipated conditions. Breaks were provided after each block and as needed.

The speed (4.5 ± 10% mph) and distance (12 ft) were chosen to appropriately bring the participant to a jog but also minimize fatigue that would occur with a greater distance and faster speed. Timing gates (SKLZ, Durham, NC, USA) placed at the start and end of the runway measured time to complete each trial and were used as a proxy variable for speed. For each trial, two trained researchers monitored the participant. A correct trial was one in which the participant achieved the following: (a) planted with the contralateral limb at the end of the runway and performed the 35–55 degree sidestep in the appropriate direction for that trial; (b) pace was consistent and average speed was in the appropriate range; (c) step time was consistent; (d) eyes were maintained on the monitor [40].

### 2.3. Standing Flanker Task

Nine participants who completed the dynamic flanker task also then completed a standing version of the flanker task. This task was developed to mimic the instructions and task demands of the dynamic flanker task but without whole-body movement. As such, it could be expected that artifacts entirely due to whole-body movement would be absent. Flanker-locked event-related potentials (ERPs) were examined between the dynamic and standing versions of the task to evaluate the ability of our processing pipeline to minimize the gait artifact while also retaining the expected ERP components (described more below). Participants were centrally positioned halfway down the 12-foot runway and stood with each heel on separate foot pedal triggers (Chronos; E-Prime 3.0; Psychology Software Tools, Pittsburgh, PA) and at a comfortable width. The stimulus presentation sequence and number of trials mimicked the dynamic task. However, rather than performing a lateral sidestep based on the direction of the middle arrow, participants used left and right foot-pedal triggers to indicate the middle arrow’s direction. Just as the dynamic flanker task, each trial began with a “Ready”? screen (1998 ms), then a cue (“Left” or “Right”) or non-cue (“XXXXX”; 1998 ms). Participants lifted their right heel in response to the “!!!”, which mimicked the first step in the dynamic flanker task, and the flanker stimulus appeared 915 ms following. Participants then lifted the heel that matched the direction of the middle arrow. Participants completed one block of 12 trials in each of the anticipated and unanticipated trials as practice followed by 8 experimental blocks of 24 trials. A correct trial was one in which participants correctly lifted their right heel to the “!!!” stimulus and the correct heel to the flanker stimulus. Trials in which the participants lifted both pedals to either the “!!!” or flanker stimulus or the incorrect pedal were coded as incorrect.

### 2.4. EEG Acquisition

Wireless EEG (250 Hz) was recorded (Explore Plus; Mentalab, Munich, Germany) from 16 Ag/AgCl gel electrodes (Fz, F3, F4, FCz, FC3, FC4, Cz, C3, C4, CPz, Pz, P9, FP1, FP2, F9, F10) and referenced online to P10. Electrodes were positioned over scalp locations within a neoprene cap (Mentalab, Munich, Germany). Movement of electrodes and wires was minimized by placing athletic tape over the electrodes, clustering the wires together to improve conductance, and a mesh skull cap. The EEG time series (250 Hz) was transmitted wirelessly (1 kHz) and synchronized with stimulus presentation timing information (E-prime 3.0; PST) in Lab Recorder v1.16.4 [41] (Lab Streaming Layer: [42]). The sum of the impedances between each individual electrode and the reference channel were kept below 20 kohm at the start of the acquisition [3]. Synchronization fidelity was monitored after acquisition for each trial based on the differences in timing onset of successive stimuli recorded in Lab Recorder and E-Prime. Any trial with packet drop of 2 samples or more was excluded, leading to a loss of an average of 1.33% of trials (*SD* = 1.81%) across participants.

### 2.5. EEG/ERP Processing

EEG processing was performed in EEGLAB 2024.0 [7] and ERPLAB 12.0 [43] in MATLAB 2024a (Mathworks, Natick, MA, USA). Since the purpose of this study was to evaluate motion artifact approaches in the EEG, three different processing pipelines were executed per participant for the dynamic flanker task. The three pipelines varied in the approach used to remove motion artifacts prior to infomax ICA. Pipeline 1 minimally corrected the continuous EEG with artifact subspace reconstruction with a modest criterion (k = 150). For Pipeline 2, the continuous EEG was corrected with a strict artifact substance reconstruction criterion (k = 15). In Pipeline 3, iCanClean with pseudo-reference channels was applied (R^2^ = 0.65). No continuous EEG rejection was performed and no channels were rejected or interpolated [1]. The processing parameters for each pipeline are first described followed by the outcomes used to compare pipelines.

#### 2.5.1. Pipeline 1—No Motion Correction

After 1 Hz (−6 db half amplitude cut off) high pass IIR Butterworth filter (12 db/oct), the EEG was minimally corrected using artifact subspace reconstruction in cleanrawdata v. 2.10 [16,17] with a conservative max acceptable 0.5 s window standard deviation (k = 150) to address any large discontinuities in the time series, and permit our use of consistent artifact-detection parameters that we could implement across pipelines. No channels or data periods were rejected, in order to maintain similarity to previous works who compared ASR vs. iCanClean, e.g., [1]. EEG was then referenced to the average of P9/P10 and then submitted to Infomax ICA (‘runica’) [7]. The P9 electrode was not submitted to ICA. Data were maintained as full rank through re-referencing to avoid the possibility of ‘ghost independent components’ that can arise from rank deficiency produced by incorrect average referencing [44]. Another way of explaining this is referencing to the average of P9/P10 did not introduce linear dependency between channels because P10 was not included in the initial dataset (e.g., re-referenced Cz = Cz − (0.5 × P9) [45]. ICLabel v1.6 [14] was used to determine each component’s likelihood of resembling brain and eye oscillations [2]. ICLabel interprets brain and non-brain sources based on an automated algorithm using crowdsourced data of nearly 6000 usable labeled EEG ICs [14]. Dipolarity of each independent component was evaluated using the dipfit v. 5.5 plugin (FieldTrip: [46], dipfit: [7]) to fit a dipole for each independent component using the template boundary element model [47]. The result of the dipole fitting is the most probable location of each component and the residual variance of each source. Residual variance quantifies the quality of each independent component based on the observed projection map and the dipole projection map [2]. ICLabel classifications (e.g., %eye), dipfit source localization (e.g., superior frontal dipole with dipfit residual variance <15%), and independent component activations were used to identify eye blink/movement components and were subsequently rejected. No component identified as muscle, channel noise, heart, or other were rejected. The EEG was then low pass filtered at 30 Hz (12 db/oct) and a bipolar horizontal eye channel created. The EEG was epoched at −4100 to 1000 ms around the onset of the flanker stimulus for correct trials and baseline corrected using a 100 ms pre-trial period (−4100 to −4000 ms). Then, a series of artifact detection steps were performed to identify any remaining artifacts after independent component correction. Eye blinks were identified as a 150 µV shift within a 200 ms window (10 ms step) at any of the 4 EOG channels. Eye movements were identified as a 50 µV shift within a 200 ms window (10 ms step) within the bipolar HEOG. Any 150 µV shift within a 150 ms window (100 ms step) at any electrode was also flagged as artifactual. Finally, any trial with voltage at any electrode exceeding −150 or 150 µV was flagged. Artifact detection was manually inspected for the presence of mechanical artifacts due to jogging. If a trial was flagged for this artifact only at one or more electrodes, the trial was retained. Trials that contained an artifact using one or more of these approaches were rejected (*M* = 4.07% trials lost, *SD* = 7.95%). Finally, all retained correct trials were averaged together for incongruent and congruent trials separately for ERP analyses. Condition differences related to anticipation and direction are not examined in this paper.

#### 2.5.2. Pipeline 2—Artifact Subspace Reconstruction

Pipeline 2 was identical to Pipeline 1 with the exception of a much stricter max acceptable standard deviation in ASR (k = 15) [15].

#### 2.5.3. Pipeline 3—iCanClean

Pipeline 3 was synonymous to Pipeline 1 with the exception of iCanClean v1.02 [1,2] with pseudo-reference signals to identify noise outside the 3–45 Hz frequency range. Noise components were identified using a 4 ms moving window and R^2^ > 0.65 because prior findings indicated these were optimal parameters for removing mechanical artifacts from mobile EEG in treadmill walking [2].

#### 2.5.4. Standing Flanker

The EEG from the standing flanker task was processed using parameters similar to Pipeline 1 (no motion correction) and epochs around the flanker onset for correct trials were segmented from −4100 to 735 ms (−200 to 0 ms baseline correction). Eye blinks were identified as a 75 µV shift within a 200 ms window (10 ms step) at any of the 4 EOG channels. Eye movements were identified as a 50 µV shift within a 200 ms window (10 ms step) within the bipolar HEOG. Any 100 µV shift within a 150 ms window (100 ms step) at any electrode was also flagged as artifactual. Finally, trials with voltage at any electrode exceeding −100 or 100 µV was flagged. Epochs with an artifact detected using one or more of these approaches were rejected. Lastly, all retained trials were averaged together for incongruent and congruent trials separately.

### 2.6. Analysis Approach and Hypotheses

Our evaluation of approaches for motion artifact removal includes two sets of analyses. First, we compared the continuous EEG from the dynamic task after preprocessing using each of the three pipelines based on (a) quality of ICA decomposition, and (b) power at the gait frequency and its harmonics. The second set of analyses compared flanker-locked ERP components between the dynamic and standing versions of the flanker task.

Pertaining to the first set of analyses, each pipeline’s performance in addressing motion artifacts was evaluated using two approaches. First, following Infomax ICA for each of the 3 pipelines, the number of independent components with ≥50% likelihood of “Brain” (ICLabel) and also high likelihood of dipolarity (dipfit residual variance ≤ 15%) were identified using a similar approach to others [2]. It was hypothesized that implementing iCanClean (Pipeline 3) would derive a larger number of dipolar “Brain” components compared to the other two pipelines [1], while implementing artifact subspace reconstruction (k = 15) in Pipeline 2 would improve the ICA decomposition relative to Pipeline 1. To test this hypothesis, a within-subjects ANOVA with pipeline as a repeated measures variable (3 levels: Pipelines 1–3) and the number of dipolar “Brain” components as the dependent variable was used. Greenhouse–Geisser was applied for potential violations of sphericity and alpha level set at 0.05 for statistical significance. Pairwise comparisons using a paired samples t-test was used to follow up a significant ANOVA test.

Second, to evaluate how each pipeline addressed power of the mechanical motion artifact, event-related spectral perturbations (ERSP) during the dynamic flanker trials were evaluated at the average of the five midline channels relative to the silent rest period prior to the trial. For each pipeline, data were first re-epoched with a 3 s window around the flanker (to derive power during movement) and cue (to derive baseline power) separately. Artifacts were detected using the same approach as outlined in Section 2.5.1 and subsequently rejected. Then, time–frequency analysis was performed (newtimef) on each participant’s epoched EEG separately using the Morlet wavelet convolution (3 0.8) and a Hanning window (200 samples, winsize 500). During early analysis, OpenAI’s ChatGPT-4 was consulted in optimizing personally derived code in newtimef and rendering some Figures. This derived averaged log power from −914 to 918 ms (1832 ms window) around the flanker onset at 113 equally spaced frequencies between 2 and 30 Hz (e.g., 2, 2.25, 2.5, 2.75, 3 Hz). This epoch was selected because jogging began with the release of the foot pedal 915 ms before the flanker and continued up to 1087 ms after flanker onset. The same approach was used to calculate average log power during the baseline period from 102 to 1934 ms (i.e., a 1832 ms window) after the presentation of the cue and prior to movement onset. Normalized power was calculated as the average log power epoched to the flanker relative to baseline’s average log power (i.e., flanker power minus baseline power). Log power was averaged between 2 and 3 Hz (capture median step frequency/trial of ≈ 2.5 Hz), 4.5–5.5 Hz (1st harmonic), and 7–8 Hz (2nd harmonic), as well as theta (4–7 Hz), alpha (8–12 Hz), and beta (13–30 Hz) [10,24]. Two participants were removed prior to the ERSP analysis because of poor accuracy and/or excessive artifacts (n = 19). A repeated measures ANOVA with pipeline (3 levels) and spectra (3 levels) evaluated how preprocessing using iCanClean and ASR prior to ICA would impact power at the gait frequency and harmonics compared to the other pipelines. A second ANOVA examined the differences between pipelines in theta, alpha, and beta frequency ranges. Greenhouse–Geisser was applied for potential violations of sphericity and alpha level set at 0.05 for statistical significance. Bonferroni-corrected pairwise comparisons using one-way ANOVAs followed up a significant ANOVA test.

Next, flanker-locked ERP components derived from the dynamic and standing versions of the flanker task were compared in two ways. First, we explored if significant congruent vs. incongruent P300 amplitude differences would be identified in both the standing flanker task and dynamic flanker task after implementing ASR (k = 15; Pipeline 2) and/or iCanClean (Pipeline 3). In flanker tasks, the incongruent condition typically evokes larger P300 ERP component amplitudes than the congruent condition [38,39] because the incongruent condition creates a conflict between multiple directional responses. The P300 reflects attentional mechanisms subsequently used for memory updating, perhaps by inhibiting task-irrelevant processing [48] and peaks 400–500 ms over central/central-parietal midline [49] but also frontocentral midline [38].

**Figure 2:**
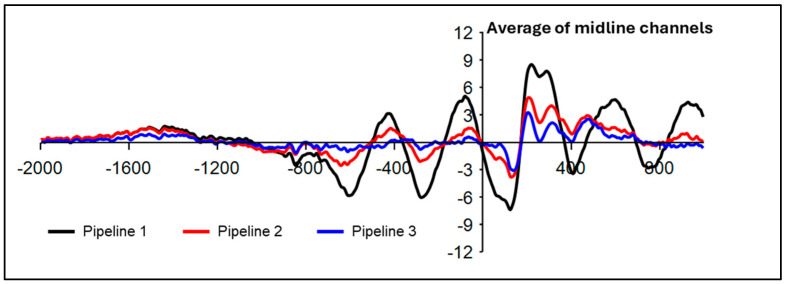
Time domain ERP plot. EEG from correct trials was time-locked to flanker stimulus onset (0 ms) and is plotted as averaged across all trial types at the average of midline channels. The presence of the jogging-related motion artifact is clear in Pipeline 1 (ASR k = 150) and Pipeline 2 (ASR k = 15), albeit to a lesser extent. Voltage (µV) is plotted on the y-axis.

Visual inspection of time–domain plots over midline channels demonstrated a P300 ERP maximum over the midline frontocentral and central electrodes. Thus, P300 congruency effects (e.g., larger amplitudes for incongruent flankers) [38,39] were examined within the dynamic flanker task and standing flanker task individually. For the dynamic flanker task, 2 × 3 repeated measures ANOVA with Greenhouse–Gessier correction tested congruent vs. incongruent condition differences in P300 peak positive amplitude and latency (400–600 ms) [49] at three midline electrodes (FCz, Cz, CPz). Bonferonni-corrected one-way ANOVAs were used to follow-up a significant interaction or main effect. Since preliminary processing indicated the robust presence of the motion artifact after implementing Pipeline 1 (Figure 2), P300 effects were not examined for Pipeline 1. Given the smaller sample size for the standing flanker task, a paired samples t-test compared P300 amplitudes and latencies between incongruent and congruent conditions at the FCz electrode. Data from the dynamic task from 3 participants were excluded because of high presence of artifacts or accuracy < 50% (n = 18).

To further evaluate similarity in ERP components between the dynamic and standing flanker tasks, N100/P200 ERP component amplitude and latencies were compared between the two tasks. Nine participants completed both the standing and dynamic flanker tasks, and one participant was excluded because of accuracy in the dynamic flanker task below 50% (n = 8). Peak amplitude and latency of the N100 (100–150 ms) and P200 (150–250 ms) were measured at Fz [50].

## 3. Results

### 3.1. Brain Source Dipolority

**Figure 3:**
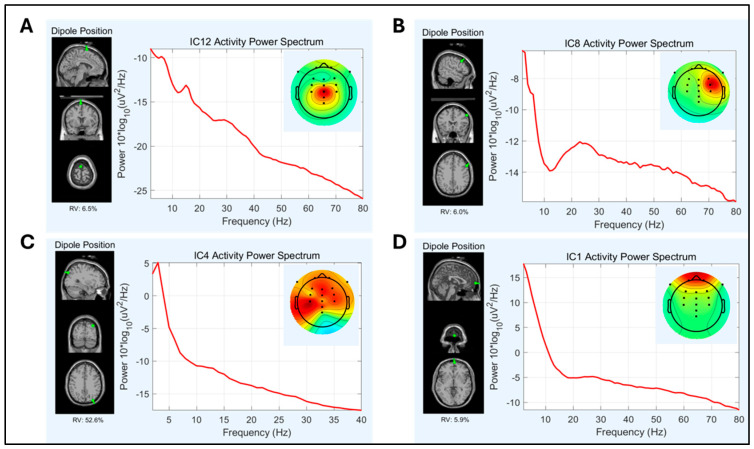
Scalp topography, power spectrum, and dipolarity localization plots for representative independent components. (**A**) Dipolar brain component: ‘brain’ = 96% (ICLabel), right postcentral gyrus (RV < 15%). (**B**) Dipolar brain component: ‘brain’ = 90% (ICLabel), right precentral gyrus (RV < 15%), beta peak at 25 Hz is typical of precentral sensorimotor processing [51]. (**C**) Non dipolar component: although ‘brain’ > 50% (ICLabel), source is not dipolar (RV = 52%). (**D**) Eye blink component: ‘eye’ = 95% (ICLabel) and dipolar source localized to superior frontal gyrus (RV < 15%). RV = residual variance. More positive voltages are reflected as brighter colors.

The number of dipolar “Brain” independent components derived from the ICA significantly varied between pipelines, *F*(1.86, 37.18) = 22.17, *p* < 0.001. Pipeline 3 produced significantly more dipolar brain components (*M* = 6.48, *SD* = 2.11) compared to Pipeline 2 (*M* = 5.38, *SD* = 1.83), *t*(20) = 2.25, *p* = 0.018, and Pipeline 1 (*M* = 3.62, *SD* = 1.75, *t*(20) = 6.87, *p* < 0.001. The difference between Pipeline 2 and Pipeline 1 was also statistically significant, *t*(20) = −4.52, *p* < 0.001. Representative components are illustrated in Figure 3.

### 3.2. Event-Related Spectral Perturbations

**Figure 4:**
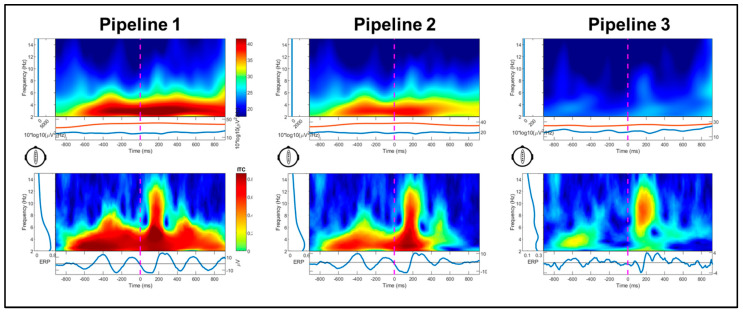
Log power (10*log_10_(µV^2^/Hz)) and inter-trial coherence changes between pipelines for a representative participant (not baseline normalized). Plots are time-locked to flanker stimulus onset (0 ms). Shown in the upper plots, in Pipeline 1 (ASR k = 150) and Pipeline 2 (ASR k = 15), large power below 3 Hz is apparent during the dynamic flanker trials with power increases for each step identified in the inter-trial coherence (lower) plots, particularly in Pipeline 1. Power below 3 Hz is reduced in Pipeline 3 and inter-trial coherence maximal during the N100/P200 ERP component range.

**Figure 5:**
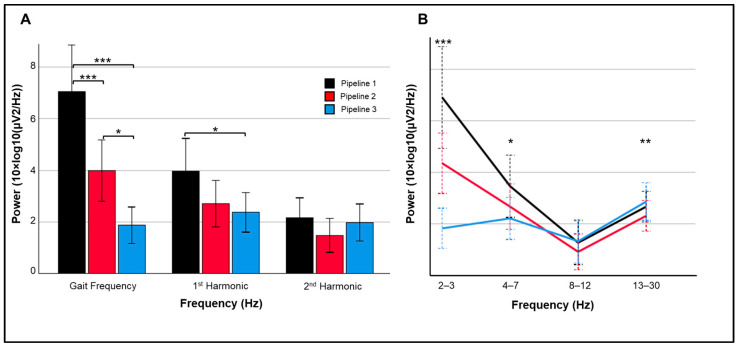
Spectral power (10×log_10_(µV^2^/Hz)) during dynamic flanker trials. (**A**) Differences between pipelines at gait frequency (2–3 Hz) and first two harmonics. (**B**) Power changes from 2 to 30 Hz for each of the 3 pipelines. Power is plotted as averaged within each frequency range and error bars represent 95% confidence intervals. *** *p* < 0.001; ** *p* < 0.01; * *p* < 0.05.

Changes in event-related power and inter-trial coherence between pipelines are illustrated for a representative subject in Figure 4. Significant main effects of pipeline, *F*(1.52, 27.36) = 12.85, *p* < 0.001, and spectra, *F*(1.36, 24.42) = 48.19, *p* < 0.001, were superseded by their interaction, *F*(1.85, 33.38) = 30.95, *p* < 0.001. Across pipelines, power was significantly greater at the mean gait frequency than its harmonics (*ps* < 0.001); power was lower at the second harmonic compared to first harmonic (*p* < 0.001). For the interaction, power varied between pipelines for the gait frequency, *F*(2, 17) = 18.95, *p* < 0.001, and first harmonic, *F*(2, 17) = 4.23, *p* = 0.031 (Figure 5A). Specifically, power between 2 and 3 Hz was significantly reduced in Pipeline 3 (iCanClean) compared to Pipeline 2 (ASR k = 15; *p* < 0.001) and Pipeline 1 (*p* < 0.001); 2–3 Hz power for Pipeline 2 was significantly lower than Pipeline 1 (*p* = 0.012). As for the 1st harmonic (4.5–5.5 Hz), Pipeline 3′s power was significantly lower than Pipeline 1 (*p* = 0.030). For Pipeline 3, power did not vary between the gait frequency or either of the two harmonics (*ps* > 0.05). For Pipelines 1 and 2, however, all pairwise comparisons between the gait frequency and the two harmonics were significant (*ps* < 0.002).

Pertaining to theta/alpha/beta frequency ranges, the main effect of spectra, *F*(1.42, 25.50) = 10.65, *p* = 0.001, and interaction between pipeline and spectra, *F*(1.86, 33.52) = 10.83, *p* < 0.001, were significant (Figure 5B). Specifically, power between pipelines significantly varied in the theta, *F*(2, 17) = 3.87, *p* = 0.041, and beta, *F*(2, 17) = 7.60, *p* = 0.004, ranges. For theta power, Pipeline 3 was significantly lower than Pipeline 1 (*p* = 0.034). For beta power, Pipeline 2 had lower power than Pipeline 1 (*p* = 0.035) and Pipeline 3 (*p* = 0.003).

### 3.3. Event-Related Potentials: Dynamic vs. Standing Flanker

#### 3.3.1. P300

**Figure 6:**
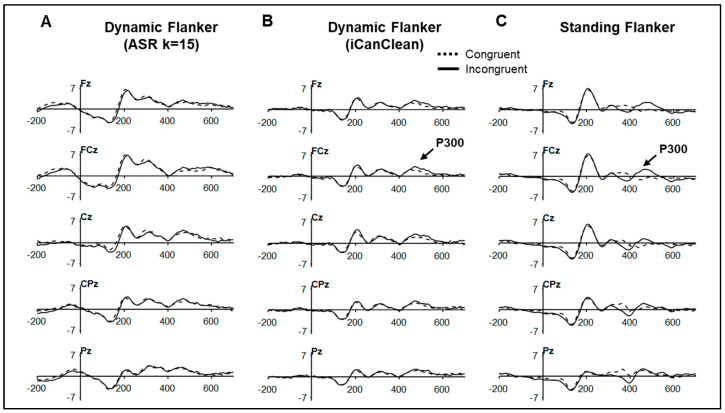
ERP plots by task at midline electrodes time-locked to flanker stimulus onset by condition. Dynamic flanker (n = 18) time–domain plots after preprocessing in (**A**) Pipeline 2 (ASR k = 15) and (**B**) Pipeline 3 (iCanClean). (**C**) Standing flanker time–domain plot (n = 8).

The P300 ERP components for the dynamic (*n* = 18) and standing (*n* = 8) flanker tasks after processing with Pipeline 2 and Pipeline 3 are shown in Figure 6 and descriptive statistics provided in Table 1. ERP components were not measured after Pipeline 1 given the presence of the large gait-related motion artifact (see Figure 2). The P300 congruency effect was identified in both tasks. For the dynamic task, after processing using Pipeline 3 (Figure 6B), the P300 amplitude was larger to incongruent than congruent trials, *F*(1, 17) = 6.56, *p* = 0.020, but stimulus congruence did not interact with electrode (*p* < 0.05). The incongruent P300 amplitude was also larger than the congruent P300 in the standing flanker task, *t*(7) = 2.23, *p* = 0.031. P300 latency did not vary between conditions for either task (*ps* > 0.10). Finally, since the P300 congruency effect was identified in the standing flanker and the dynamic flanker tasks (Pipeline 3), the presence of this effect was also examined in the dynamic flanker task after preprocessing using Pipeline 2 (ASR k =15) using the same repeated measures ANOVA model as Pipeline 3. Although the P300 appears at the expected time course in Pipeline 2’s time–domain plot (see Figure 6A), its amplitude did not vary between incongruent vs. congruent conditions, *F*(1, 17) = 0.616, *p* = 0.443.

#### 3.3.2. N100/P200

Descriptive statistics for N100 and P200 amplitude and latency for the subsample who completed both dynamic and standing tasks (n = 8) as well as the full sample from the dynamic task (n = 18) are provided in Table 2 and plotted in Figure 6. N100/P200 metrics from the full sample are provided for descriptive purposes; only the components for the subsample who completed both dynamic and standing flanker tasks were compared (n = 8). The amplitude of the P200 was larger in the standing task vs. dynamic task, *t*(7) = −3.01, *p* = 0.010. N100 amplitude/latency and P200 latency did not vary between tasks.

## 4. Discussion

The purpose of this study was to evaluate commonly used artifact removal preprocessing techniques for reducing the motion-related artifact in the EEG during running. We focused on comparing iCanClean with pseudo-reference noise channels to artifact subspace reconstruction (ASR) because of recent work showing that preprocessing EEG during simulated walking using iCanClean, with dual-layer mechanically coupled noise sensors, reproduces EEG closest to ground-truth signals compared to ASR [1] and its recent applications in human walking [2,23]. However, ASR has been shown to improve the quality of ICA decompositions during whole-body movements compared to minimal processing [52]. We sought to determine the performance of iCanClean and ASR when fewer electrodes are available and without dedicated noise sensors (for iCanClean).

At present, there is no consensus on how mobile EEG scientists should evaluate the presence of motion-related artifacts in walking or running studies nor agreed upon standards for determining how preprocessing pipelines have sufficiently minimized the artifact [53]. Therefore, EEG scientists are challenged with optimizing processing pipelines that balance the reduction in the mechanical artifact while minimizing the potential for overcleaning and developing ways to assess these techniques. In this study, we used converging and multiple time–frequency and time–domain approaches to characterize the running-related mechanical artifact and how ASR and iCanClean addressed the artifact in the EEG. To evaluate the running-related EEG, we developed a dynamic (i.e., whole-body jogging) version of an adapted flanker task which allowed for the comparison of brain source dipolarity, spectral power differences, and flanker-locked ERP components based on motion artifact removal approaches. Finally, the extent to which any preprocessing tool, particularly those designed to correct motion artifacts, under- vs. overcleans the EEG requires an understanding of the EEG’s ground-truth signal, which cannot be reconstructed with certainty from human scalp EEG. Thus, we developed a standing flanker task, which was designed to mirror the dynamic flanker task without the running-related artifact, to serve as a standard for comparing ERP-components after motion artifact removal.

Our first method for evaluating motion artifact removal approaches was to submit the EEG processed using ASR (k = 15) and iCanClean to Infomax ICA and compare the dipolarity of ‘Brain’ independent components resulting from the ICA, similar to others [2]. We identified those independent components with spectral properties classified as ‘Brain’ from ICLabel and dipolar with residual variance < 15% identified by dipfit source localization. In support of our first hypothesis, we found that preprocessing the EEG from the dynamic flanker task using iCanClean produced more dipolar brain components compared to ASR with k = 15 threshold, whereas the latter outperformed the pipeline with minimal cleaning (ASR k = 150). Although iCanClean has been shown to reproduce EEG that are most like simulated ground-truth signals as compared to ASR [1], this is the first study (to our knowledge) to compare their brain source dipolarity during overground whole-body movement. At a minimum, this study and others have shown that ASR with a modest criterion (e.g., k = 10–30) improves independent component dipolarity. For example, from dual-task EEG recorded during skateboarding, ASR with a similar standard deviation cutoff (k = 20) produced more ‘Brain’ independent components than processing without ASR [52].

It is possible that implementing ASR with different parameters or a more conservative threshold criterion might produce different results. For example, we did not implement automatic channel rejection in clean_rawdata [1], which might have negatively impacted ASR in this study. Pertaining to the SD threshold parameters in ASR, some have recommended a k value of 20–30 because with more aggressive k values fewer dipolar brain components were identified and the power of those brain components were reduced below 50%, for example [19]. However, EEG recorded in their study was during simulated driving, not locomotion, and so stricter k values may be more appropriate. For example, ASR with standard deviation cutoffs of 10 or more have been shown to produce the most reproducible and dipolar independent components during human walking [22]. Some have also shown that implementing ASR with strict k thresholds substantially reduce signal variance [19], which may reduce EEG signal fidelity in experimental designs. Given these recommendations, and the likelihood of larger artifacts produced during running than walking, we chose a modest k = 15 criterion.

Although k values between 10–30 have been recommended [19,22] the limitations of using ASR with standard deviation of more than 10 has been highlighted in a recent paper [20] because of ASR’s mechanism for selecting the reference data. ASR’s algorithm forms the basis for determining data periods that are artifactual and thus interpolated from the reference data. In high-density EEG recorded during juggling, the authors recently showed that ASR’s original algorithm (implemented in the current study) has a tendency for high-amplitude artifacts to ‘pass under’ the parameters required for exclusion from the reference period. This is because up to 7.5% of channels with outlier values within a data segment are permitted in the reference data; it is possible for approximately 0% of data to be included in the reference period when a 0% outlier criterion is used [20]. If the artifactual data permeate into the reference data, the Gaussian distribution of RMS z-scores is strongly positively skewed and biases the standard deviation which forms the basis of the k threshold [20]. This is particularly more likely when ASR’s sampling window length is longer than the high-amplitude motion artifacts. The study’s results showed that 43% of the EEG time series was included in the reference data (based on the ASR algorithm reviewed in the introduction), including many high-amplitude artifacts, which were eliminated when using new algorithms which do not use sliding window RMS values. Thus, the authors conclude that ‘counterintuitively large’ SD thresholds reported in the literature (e.g., k = 10–30) have been used because the skewness in the Gaussian distribution misrepresents the true distribution of the signal variance and thus biases the k criterion. They proposed two new ASR algorithms that correct for this bias, avoid high-amplitude artifacts from breaching the reference data, and, using these new variants of ASR, propose that k values as low as 3–5 may be most appropriate [20].

We included a 10 min resting state (eyes-open) baseline for all participants for use in calculating the reference period. This reduces the likelihood that high-amplitude artifacts were included in the reference period [20]. In preprocessing using ASR on several subjects, visual inspection of the data in the time–domain also suggested that stricter k values ‘overcleaned’ the EEG, which is why k = 15 was chosen. Furthermore, others suggested that k values below 10 may negatively impact the reproducibility of independent components [18]. Given proposed versions of ASR which address limitations in the extraction of the reference data [20], if these are implemented in EEGLAB, we recommend that future studies test these proposed algorithms using stricter k values in line with recent recommendations [20]. It is possible that this approach to ASR would improve the reconstruction of the EEG time series which may limit the need of reference noise signals, such as those implemented in iCanClean. Future work may consider evaluating if a more conservative k parameter and the proposed algorithms to ASR would produce more dipolar ‘Brain’ independent components.

We also used a time–frequency approach to compare power between processing pipelines at the step frequency and its first two harmonics [3]. Gwin and colleagues [3] showed that a combined channel- and ICA-based artifact removal approach eliminated the mean spectral power within the range of the step frequency and the first two harmonics during walking, which was reduced, but not eliminated, during running. In the present study, we examined spectral power during the movement phase of each trial during the dynamic flanker task relative to a non-movement baseline period. Preprocessing with iCanClean reduced power of at the step artifact frequency compared to processing with ASR (k = 15) and minimal processing (ASR k = 150). Similarly to the dipolarity results, the power at the step frequency was reduced after processing with ASR k = 15 (Pipeline 2) than ASR k = 150 (*p* < 0.001). Power at the gait frequency and its harmonics did not vary after processing with iCanClean, whereas power significantly decreased from the gait frequency to the first and second harmonics after processing with ASR (*ps* < 0.001).

These findings paired with visual inspection of the artifact for each pipeline (Figure 2) suggest that iCanClean with pseudo-reference signals reduces the power of the gait artifact, although it is possible the artifact is not eliminated, and it is unknown how the bandstop filter implemented in iCanClean with pseudo-reference signals reduces spectral power of the ground-truth signal. In an attempt to address this uncertainty, we compared flanker-locked ERP components derived from the dynamic flanker task (after processing with ASR k = 15 and also iCanClean) to those evoked from the standing flanker task which had no motion artifact. This is because endogenous ERP components, such as the P300, are thought to be derived from increases in theta and delta spectral power [54,55] which may overlap with the frequency of the step artifact.

Processing with iCanClean reduced spectral power in spectral range of high delta/step artifact (2–3 Hz) and first harmonic (4.5–5.5 Hz), the latter of which contributed to the lower power in the theta range (4–7 Hz). Processing with ASR (k = 15), although reducing power at the step frequency compared to minimal processing, did not impact power at the first harmonic or theta ranges. It is unknown if processing with ASR (k = 15) or iCanClean with pseudo-reference signals (bandstop 3–45 Hz) may be artificially reducing dipolar brain activity in the delta and/or theta frequency ranges. One potential way to evaluate any ‘overcleaning’ produced by ASR or iCanClean is to examine P300 ERP components derived from the standing flanker task (Figure 6C) and dynamic flanker task after processing with ASR (Figure 6A) and iCanClean (Figure 6B).

First, it does not appear that preprocessing with iCanClean affected the P300 ERP; its latency is at the expected time course compared to the P300 derived from the standing flanker task and the larger P300 amplitude to incongruent trials was identified. After processing with ASR (k = 15) in Pipeline 2, the P300 can be visually identified in Figure 6A but an amplitude difference between incongruent and congruent conditions was not identified.

Finally, since walking is characterized by alpha and beta suppression [24,25], we considered how processing the EEG with ASR and iCanClean differentially impacted alpha and beta power during the dynamic flanker task. We found that beta power (13–30 Hz) was lower after processing with ASR (k = 15) compared to minimal processing (ASR k = 150) and processing with iCanClean, whereas alpha power did not vary between artifact removal approaches. However, the alpha/beta suppression that occurs during walking is commonly localized and measured from sensorimotor independent components only, whereas we used a channel-based approach (average of midline channels) to characterize the motion artifact. Furthermore, alpha and beta spectral changes during the gait cycle have been identified using both ASR (k = 7) and iCanClean using 128-electrode EEG [24]. Thus, when larger electrode montages are available, it may be that ASR with a strict cleaning threshold may suffice to minimize the motion-related artifact and capture expected alpha/beta power changes during the gait cycle, even at faster speeds [24]. Future research may also leverage deep learning structures (e.g., convolutional neural networks) for classifying electrocortical states during the gait cycle, similar to what has been applied to cognitive state classification [56].

The present study has limitations. First, we sought to evaluate motion artifact removal approaches from a low-density array (16-channels) because such configurations are more feasible to implement than high-density systems [57], and may be of interest for future clinical translations, such as in examining cognitive-motor processing involved in recreational injury prevention. It is possible that our choice of fewer electrodes impacted source localization of independent components after Infomax ICA which would be minimized in future studies with more electrodes. High-density arrays of 120-channels or more are typically used to construct time–frequency decompositions for individual EEG sources (resulting from ICA), such as investigations of alpha/beta suppression localized to sensorimotor sources [24,25]. Although 35-channels may suffice to identify major electrocortical dynamics changes during locomotion, the correlation between independent component activation from low-density montages (e.g., 25-electrodes) and high-density montages during is known to decrease with fewer channels [58]. It is also possible that having more channels would improve the performance of both iCanClean and ASR. For example, when using dedicated noise sensors in dual-layer iCanClean, the number of dipolar ‘brain’ independent components marginally increased from 16 to 120 noise sensors [2]. Few preliminary studies shown ASR to perform well in handling typical artifacts in low-density scenarios [27,28] but have not been investigated with more burst-like artifacts such as those that occur from whole-body movement in the present study. Research using filter optimization algorithms also suggests that an IIR Chebyshev filter best preserves the beta signal, by reducing average power, and so it is possible that our choice of the IIR Butterworth filter impacted beta power [59].

Furthermore, ICLabel’s component categorization is trained on average-referenced data whereas we chose to re-reference the EEG in the present study to the average of P9/P10 because of the low-density array. Although this may have impacted the characterization of independent components and their dipolarity, the choice of reference was identical in the different pipelines. We recommend future work to evaluate the difference in brain source dipolarity between motion artifact removal approaches in a higher-density array.

Second, our study did not include a comprehensive comparison of motion artifact removal tools and limited in its evaluation of ASR and iCanClean and select parameters. For example, it is possible that implementing ASR with a more strict or conservative k criterion may have produced more or less dipolar ‘brain’ independent components. However, we chose a standard deviation threshold (k = 15) that may seem strict compared to non-locomotion studies [19] but is more conservative than what is typically implemented in studies of human walking, such as k = 7 [24] and k = 10 [22]. Pertaining to iCanClean, we chose those parameters that were shown to produce the most dipolar brain independent components in human walking [2] but we did not perform a full parameter sweep. Recent findings indicate that preprocessing EEG during walking with dedicated noise sensors (‘dual layer’) is a robust approach to best reconstructing the otherwise artifact-free EEG [1,2,23,24]. However, to our knowledge the use of pseudo-reference noise channels from single-layer EEG has not been implemented in iCanClean, and so was the focus of the present study. Thus, muscle artifacts may have been more frequent in our studies than others that have dedicated EMG sensors, such as on the bilateral sternocleidomastoid and trapezius muscles [23]. Future studies may consider comparing the performance of iCanClean in reducing the motion-related artifact with and without dedicated noise sensors.

Third, the dynamic flanker task in the present study was designed to simulate the cognitive–motor interference encountered during recreational activity and allow for the evaluation of ERPs between processing pipelines. Thus, the EEG was collected during overground running rather than on a treadmill [3,10,23,24]. Although speed was monitored and approximated between 4.05 and 4.95 mph based on timing gates placed at the start and end of the runway, the frequency of the motion artifact is more consistent when speed can be controlled on a treadmill. Consequently, we chose to evaluate the motion-related artifact as the average power between 2 and 3 Hz because it encompasses the median step frequency but also captures slight and allowable individual differences in speed. Finally, although the present study inertial measurement units were used to measure tibial accelerations, these were not temporally synced with the EEG. Therefore, the evaluation of the motion-related artifact was time-locked to the onset of the flanker stimulus and encompassed the entire movement period before the flanker (915 ms) and a similar post-flanker temporal window. Consequently, event-related spectral perturbations during the dynamic flanker task began with the swing phase of the gait cycle (i.e., the step off the trigger pedal), rather than epoching the EEG time-locked to heel strikes. In future studies, we look forward to integrating heel strike measurements with the EEG time series.

## 5. Conclusions

Identifying and correcting the motion-related artifact from the EEG during human running is a challenge for investigators seeking to characterize brain–biomechanics relationships in healthy and injured populations during recreation. We collected 16-channel mobile EEG from healthy, active young adults during a novel dynamic version of the flanker task to evaluate motion artifact removal approaches based on the power of the gait artifact and flanker-locked ERPs. We found that preprocessing using iCanClean with pseudo-reference noise channels produced the most dipolar brain components and reduced the motion-related artifact compared to artifact subspace reconstruction (with k = 15), whereas ASR outperformed the minimally processed EEG. Additionally, we introduced a standing version of the flanker task as an approximation of the ‘ground truth’ flanker-locked ERPs in absence of the motion artifact. We found that the time course of the N100, P200, and P300 ERPs was similar between the standing flanker task and dynamic flanker task after processing with iCanClean. Finally, the P300 congruency effects identified in the standing flanker task were only identified in the dynamic flanker task after processing with iCanClean. Processing EEG using iCanClean during human running may be an effective approach for reducing the motion-related artifact and evaluating cognitive-motor ERPs. We recommend future studies evaluate the iCanClean tool for motion artifact removal during running with a larger density array.

## Figures and Tables

**Table 1:** P300 amplitude (400–600 ms) congruency effect at FCz.

	Congruent (M ± SD)	Incongruent (M ± SD)	Test Statistic
Dynamic flanker ^	3.71 ± 1.32	4.45 ± 1.73	*t*(17) = −2.0, *p* = 0.031
Standing flanker	2.48 ± 2.72	3.81 ± 2.34	*t*(7) = −2.22, *p* = 0.031

*^* Pipeline 3 (iCanClean).

**Table 2:** N100 and P200 (Fz) amplitude and latency differences between tasks.

	Dynamic (n = 18)	Dynamic (n = 8)	Standing (n = 8)	*t(7)*
N100 latency	130.89 ± 14.76	135 ± 16.53	129.50 ± 12.82	1.05
N100 amplitude	−4.13 ± 2.30	−4.22 ± 2.27	−5.19 ± 2.85	1.05
P200 amplitude	4.84 ± 2.58	4.51 ± 2.49	7.51 ± 4.05	−3.01 *
P200 latency	208.89 ± 20.74	204.5 ± 25.61	208 ± 18.27	−0.50

* p < 0.05; Dynamic task data from Pipeline 3 (iCanClean).

## Data Availability

The datasets presented in this methodological article are part of an ongoing study and will be made available following publication of its substantive work. Requests to access the datasets should be directed to the corresponding author.

## Abbreviations

The following abbreviations are used in this manuscript:

EEG	Electroencephalography
ERP	Event-related potential
ICA	Independent components analysis
ASR	Artifact Subspace Reconstruction
EOG	Electrooculography
HEOG	Horizontal Electrooculography
